# Audiovisual temporal recalibration occurs independently at two different time scales

**DOI:** 10.1038/srep14526

**Published:** 2015-10-12

**Authors:** Erik Van der Burg, David Alais, John Cass

**Affiliations:** 1 School of Psychology, University of Sydney, Australia; 2 School of Psychology, Western Sydney University, Australia

## Abstract

Combining signals across the senses improves precision and speed of perception, although this multisensory benefit declines for asynchronous signals. Multisensory events may produce synchronized stimuli at source but asynchronies inevitably arise due to distance, intensity, attention and neural latencies. Temporal recalibration is an adaptive phenomenon that serves to perceptually realign physically asynchronous signals. Recently, it was discovered that temporal recalibration occurs far more rapidly than previously thought and does not require minutes of adaptation. Using a classical audiovisual simultaneity task and a series of brief flashes and tones varying in onset asynchrony, perceived simultaneity on a given trial was found to shift in the direction of the preceding trial’s asynchrony. Here we examine whether this inter-trial recalibration reflects the same process as prolonged adaptation by combining both paradigms: participants adapted to a fixed temporal lag for several minutes followed by a rapid series of test trials requiring a synchrony judgment. Interestingly, we find evidence of recalibration from prolonged adaptation and inter-trial recalibration within a single experiment. We show a dissociation in which sustained adaptation produces a large but decaying recalibration effect whilst inter-trial recalibration produces large transient effects whose sign matches that of the previous trial.

Physical events may produce signals activating multiple sensory modalities. Provided these signals activate the brain in close temporal proximity they may interact^1^,^2^,^3^,^4^,^5^,^6^. For example, search for a visual target among distractors may be facilitated when changes in target luminance are sychronised with an auditory signal^7^, and conversely, it is easier to understand speech in noisy environments when we observe the lip-movements of the speaker^8^. Typically, multisensory benefits are optimal when the unimodal signals are perceived simultaneously and decline with increasing asynchrony^9^,^10^,^11^. This presents a challenge because in natural scenes, although audiovisual signals may originate from a single source, they are likely to activate the brain’s unisensory cortices asynchronously due to different propagation speeds for sound and light as well as different neural transduction and latency times.

Interestingly, the brain appears to compensate for multisensory asynchrony. Psychophysical experiments show that prior exposure to asynchronous audiovisual events shifts the point of perceived simultaneity for subsequent audiovisual events in the direction of the preceding asynchrony^12^,^13^. For example, if one adapts to a flash followed by a tone 100 ms later, one will subsequently perceive audiovisual pairs with the same temporal lag as being more synchronous. Studies examining temporal recalibration have typically employed several minutes of adaptation to induce the effect (see e.g.^12^,^13^,^14^,^15^,^16^,^17^,^18^,^19^,^20^,^21^; and see^22^, for a nice demonstration about how recalibration builds up). Recently, however, it was discovered that recalibration can occur extremely rapidly, with Van der Burg, Alais and Cass^23^ reporting large temporal recalibrations without prolonged adaptation. In a classical audiovisual simultaneity judgment (SJ) task, they demonstrated that the point of subjective simultaneity (PSS) for a given audiovisual pair shifts in the direction of the modality leading on the preceding trial. This indicates that temporal recalibration occurs far more rapidly than previously thought and may only require exposure to a single, brief asynchrony.

## Experiment

**Figure 1:**
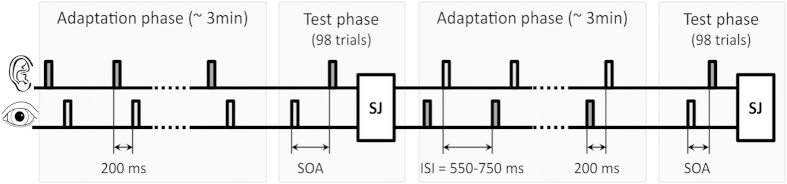
Setup of one experimental session. Each session consisted of two periods of prolonged adaptation (*adaptation phase*), each followed by a *test phase*. During an adaptation procedure, participants were presented with a series of 235 abrupt luminance-defined flashes, each presented with a brief tone ‘pip’ with a fixed temporal lag, for approximately 3 minutes. During one adaptation phase, the auditory signal always led by 200 ms, whereas in the other the auditory signal lagged by 200 ms (or vice versa). The modality order in the adaptation phase (auditory lead or visual lead) was counterbalanced across participants. The inter-stimulus-interval (ISI) was randomly jittred over a narrow range (550–750 ms) and averaged 650 ms. In the test phase, participants were presented with 98 trials of asynchronous audiovisual pairs varying across a range of ±SOAs, and participants were required to make a synchrony judgment (SJ) to each pair. In total participants completed four sessions.

In this study we examine whether the recalibration effects arising from the prolonged and inter-trial paradigms are common or independent processes. To examine this we used a standard prolonged period of asynchronous audiovisual adaptation (3 mins, see *adaptation phase*, Fig. 1) followed by a sequence of 98 audiovisual test trials of variable positive and negative SOAs, each requiring a synchrony judgment (‘synchronous’ or not, see *test phase*, Fig. 1).

During the prolonged adaptation phase participants were presented with a series of 235 abrupt luminance-defined flashes, each accompanied by a brief tone ‘pip’ at a fixed temporal lag, for approximately 3 minutes. The interval between the audiovisual pair was always 200 ms, but during one adaptation phase the sound would lead while in the other vision would lead (with the adapting order counterbalanced over subjects). During the test phase, participants were presented with a series of 98 flash/tone pairings across a range of positive and negative SOAs and judged whether each pairing was synchronized or not. Following prolonged adaptation we would expect the PSS to be shifted in the direction of the adapted audiovisual lag, an effect which has been found to decay over time and return to baseline^24^. During the test phase we expect an inter-trial effect^23^ whereby the PSS on any given trial rapidly recalibrates in the direction of the leading modality on the preceding trial. It is unclear however, how this rapid recalibration effect relates to the recalibration resulting from prolonged adaptation. One possibility is that inter-trial recalibration would ‘overwrite’ the effect of prolonged adaptation. Alternatively, the processes may interact in other ways such that the magnitude and sign of recalibration at one scale affects the magnitude and sign at the other scale. For example, inter-trial recalibration could be weak when prolonged recalibration is strong and vice versa. Alternatively, two forms of recalibration may operate independently at both time scales. If so, we expect both the sign and magnitude of PSS shifts due to prolonged and inter-trial adaptation to combine additively across trials.

## Methods

### Participants

Ten human participants (eight female; mean age: 20.8, ranging from 19 to 29 years) participated in the present experiment. All participants were naïve as to the purpose of the experiment and were paid $AU 20 per hour for their participation. Informed consent was obtained from each participant after the nature of the study was explained to them. The research was approved by the Ethics Committee of the University of Sydney. The experiments were conducted according to the principles laid down in the Helsinki Declaration.

### Stimuli and apparatus

The experiment was programed and run using Eprime software. Participants were seated in a dimly lit room, and the CRT monitor was viewed from approximately 80 cm. The tones were delivered over Sennheiser headphones (HD380 pro). A white fixation dot was presented on a black screen throughout the experiment. For both adaptation and testing, the visual stimulus was a white ring (radius 2.6°; width 0.4°) surrounding the fixation dot, and the auditory stimulus was a pure tone (500 Hz; 44.1 kHz sample rate).

### Procedure and design

Each session involved two adaptation procedures, each followed by a test procedure. Adaptation consisted of 235 audiovisual events that were asynchronous by a fixed temporal lag of 200 ms, either vision first or audition first, in a counterbalanced order across participants. The auditory and visual stimuli were each 50 ms in duration and the ISI between successive adapting stimuli averaged 650 ms, varying randomly between 550 and 750 ms in 50 ms steps to avoid predictable rhythmicity. The adaptation procedure lasted ~200 seconds (235 trials × 850 ms) and participants maintained fixation on a central white dot that was present throughout the experiment. The test phase began with a white fixation dot for 1000 ms after which a rapid series of test stimuli (white ring combined with the tone) was presented. The tone either preceded or followed the ring’s onset by a SOA drawn randomly from the set (0, 64, 128, 256, 512 ms). The task was to judge whether the onset of the ring and tone was synchronous or not by pressing the 1- or 0-key, respectively. Whereas the test tone was presented for 50 ms, the test ring remained on the screen until the unspeeded response was made see also^23^. A test phase contained 98 trials, comprising 14 presentations of the 128, 256 and 512 ms SOA conditions and 28 presentations of the 0 and 64 ms SOA conditions. Participants each completed four sessions and once the session began breaks were not permitted.

### Analyses

To reveal the initial effect of prolonged adaptation we fitted a Gaussian distribution to the first 50 synchrony judgments, with mean, bandwidth and amplitude as free parameters. As participants completed four sessions, pooling the first 50 trials in each made a total of 200 trials for fitting. The mean of the best-fitting Gaussian was taken as the estimate of PSS, and this was done for both modality orders during adaptation to show the separate effects on PSS of prolonged adaptation to vision-leading and to audition-leading stimuli. A negative PSS indicates that audition leads vision, whereas a positive PSS indicates that vision leads audition. To reveal the rapid inter-trial recalibration effect we did an inter-trial analysis on the first 50 synchrony judgments, again pooling over sessions to obtain 200 trials. This inter-trial analysis involved allocating the response on a given trial to one of two categories based on whether the preceding trial was a visual-lead or auditory-lead trial. Gaussian distributions were then fit to each category of ~100 trials to reveal how the PSS on a given trial depended on the sign of the previous trial’s asynchrony. Using this procedure, two Gaussian’s were fit (one for each sign of preceding SOA) to the data obtained following prolonged initial adaptation to vision-lead stimuli, and two were fit to the data obtained following prolonged initial adaptation to auditory-lead stimuli, making a total of four Gaussians.

To reveal the time-course of the two adaptation effects we moved our window of analysis in one-trial increments from trials 1–50, 2–51, 3–52, etc. until the final (98^th^) trial was reached, making a total of 49 time points. We repeated the analyses of both effects at each point and plotted them as a function of time. That is, for a given window, we calculate the average absolute duration of the response since the offset of the preceding adaptation procedure.

## Results

**Figure 2:**
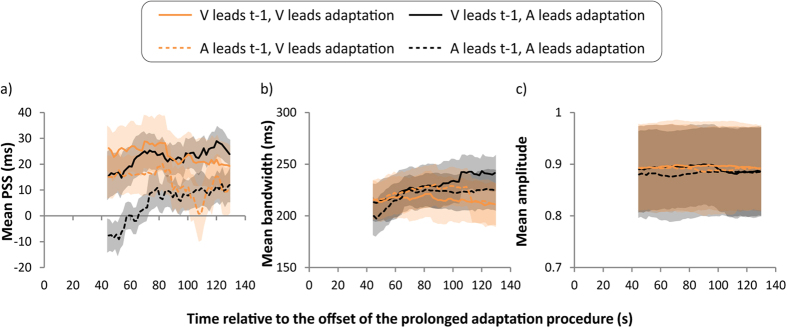
Mean parameter values from the best-fitting Gaussians fitted to synchrony judgments during the test phase. (**a**) Group mean PSS as a function of time relative to adaptation offset for each modality order on the preceding trial and for each modality order during the prolonged initial adaptation procedure. Here, a negative PSS indicates that audition leads vision, whereas a positive PSS indicates that vision leads audition. (**b**) Group mean band width as a function of time relative to adaptation offset for each modality order on the preceding trial and for each modality order during the prolonged initial adaptation procedure. (**c**) Group mean amplitude as a function of time relative to adaptation offset for each modality order on the preceding trial and for each modality order during the prolonged initial adaptation procedure. All error bars represent ± 1 s.e.m.

ANOVAs were conducted on the distributions’ *PSS*, *Bandwidth* and *Amplitude*, with *Modality order on t-1*, *Modality order during adaptation* and *Time since adaptation offset* as within subject variables. Alpha was set to .05, and *p* values were Huynh-Feldt corrected to deal with sphericity violations. Note that for the inter-trial analyses the first trial of each block was necessarily excluded. Figure 2 illustrates the mean PSS, bandwidth and amplitude of the best-fitting Gaussians plotted as a function of time since the offset of the initial prolonged adaptation period. There are four curves in each panel: for each modality order of prolonged initial adaptation (A-lead or V-lead), there are two kinds of inter-trial order (A-lead or V-lead on the preceding trial).

### Point of subjective simultaneity (PSS)

**Figure 3:**
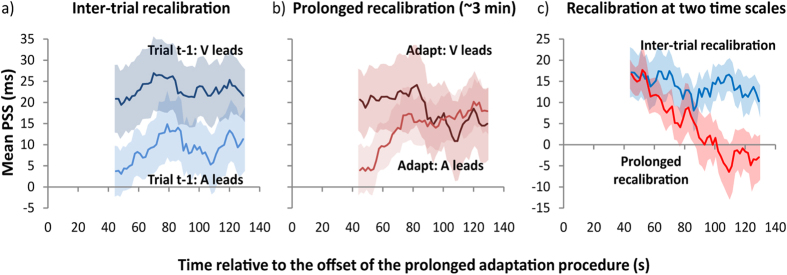
Recalibration at two different time scales. (**a**) Inter-trial adaptation effects: Mean point of subjective simultaneity (PSS) as a function of time since offset of prolonged adaptation plotted separately for both types of modality order on trial t-1 (i.e., vision led or audition led). Note that 0 s indicates adaptation offset and the first data point is located at the temporal midpoint of the 50 trials in the moving window of analysis. The light blue curve shows PSSs for trials in which audition led on trial t-1 and the dark blue curve shows PSSs for trials in which vision led on trial t-1. (**b**) Effects of prolonged adaptation: Mean PSS as a function of time since offset of prolonged adaptation plotted separately for both types of modality order during adaptation. The light red curve shows PSSs for trials in which audition led vision by 200 ms during adaptation, and the dark red curve shows PSSs for trials those trials in which vision led audition by 200 ms during adaptation. (**c**) Inter-trial and prolonged recalibration as a function of time since adaptation offset. The blue curve plots the rapid recalibration effect (i.e., ΔPSS = PSS vision leads on trial t-1—PSS audition leads on trial t-1). The red curve plots the prolonged recalibration effect (i.e., ΔPSS = PSS vision leads during initial adaptation—PSS audition leads during initial adaptation). In Fig. 3, a negative PSS indicates that audition leads vision, whereas a positive PSS indicates that vision leads audition. All error bars represent ± 1 s.e.m.

We observed a strong inter-trial recalibration effect as the PSS was significantly shorter (9 ms) when audition led on the preceding trial than when vision led on the preceding trial (23 ms), *F*(1, 9) = 12.9, *p* = .006. The interaction between modality order on trial t-1 and time since adaptation offset was far from significant, *F*(47, 423) = .8, *p* = .538, indicating that the inter-trial recalibration effect remained constant over the test phase of the experiment (see Fig. 3a). The blue line in Fig. 3c illustrates the inter-trial recalibration effect (i.e., ΔPSS = PSS for vision-lead on trial t-1—PSS for audition-lead on trial t-1) and shows that this effect did not depend on the time since adaptation offset. Turning to analysis of prolonged adaptation, the main effect of modality order during the initial prolonged adaptation phase (see Fig. 3b) failed to reach significance, *F*(1, 9) = 1.3, *p* = .290. The two-way interaction, however, between time since adaptation offset and modality order during initial adaptation was significant, *F*(47, 423) = 5.0, *p* = .010. This interaction was further examined by pair-wise two-tailed t-test for each bin. For the first 13 bins (up to 66 seconds after adaptation offset), the PSS was significantly shorter when audition led during initial adaptation than when vision led during initial adaptation (bins 1–5: *ps* < .005; bins 6–13: *ps* < .05). For all subsequent bins, none of the t-tests were reliable (*ps* > .115). To correct for multiple comparisons, we conduct false discovery rate (FDR)^25^ correction on the resulting *p* values. After correction, only the first five bins (up to 52 seconds after adaptation offset) were considered as reliable prolonged adaptation effects. The red line in Fig. 3c illustrates the prolonged recalibration effect (i.e., ΔPSS = PSS for vision leading during adaptation phase—PSS for vision leading during adaptation phase), and clearly illustrates, in stark contrast to the sustained effect of inter-trial recalibration, that the effect of prolonged adaptation decreased over time and disappeared. Finally, although it is clear that the PSS depends on both the modality order in the preceding trial and on the modality order during the initial adaptation procedure, it is important to note that these different recalibration effects were additive and independent of each other, as all other effects were not significant (*Fs* < 1.6, *ps* > .213).

### Bandwidth (SD) and amplitude

Figure 2b,c illustrate the bandwidths and amplitudes of the Gaussian fits to the synchrony distributions as a function of time since adaptation offset for the modality order on each preceding trial and for the modality order during the prolonged initial adaptation phase, respectively. The ANOVA on bandwidth yielded a reliable main effect of time, *F*(47, 423) = 3.6, *p* = .046, with bandwidths increasing sligthly over time. All other effects were not significant (*Fs* < 2.5, *ps* > .143). The ANOVA on amplitude yielded no significant effect (*Fs* < 2.3, *ps* > .162).

## General Discussion

In the present study we found evidence that temporal recalibration (shifts in PSS due to prior asynchronous exposure) can operate simultaneously at multiple time scales. The more prolonged of these recalibration processes results from prolonged and repeated exposure to a given audiovisual asynchrony and decays slowly, returning to baseline a minute or so after the three-minute adaptation procedure ceases^24^. The other process (inter-trial recalibration) occurs rapidly, with its magnitude and sign depending on the modality order of the preceding trial. Whereas prolonged recalibration causes a strong aftereffect that lasts approximately one minute, rapid recalibration varies from trial to trial, determined by the order of the audio-visual stimuli on the preceding trial. Although this is not the first study to show prolonged recalibration see e.g.^12^,^13^ or inter-trial recalibration^23^,^26^,^27^, we are the first to show that both recalibration effects can occur concurrently in a single experimental paradigm.

We find that although the magnitude of prolonged recalibration decreased following initial exposure, this had no effect on the either the magnitude or sign of inter-trial recalibration. On the face of it the absence of any statistical interaction seems to imply the existence of separate independent processes. This interpretation, however, rests on the assumption that the maximum recalibration effect we observe represents a saturated (i.e., fully adapted) state of the prolonged recalibration process, which, if at ceiling, ought to be incapable of further inter-trial shifts in PSS in the same direction as the prolonged lag. If, however, the prolonged recalibration process were not fully adapted, it is conceivable therefore that both the prolonged and rapid recalibration we observe may result from a common process. At present our results are unable to differentiate between these possibilities. Future studies, may therefore consider systematically increasing the period of prolonged adaptation to ensure saturation has been reached. In a single-mechanism framework, there should be no inter-trial adaptation effect when adaptation is fully saturated.

What might be the functional purpose of rapid and prolonged recalibration? Rapid inter-trial recalibration makes sense in a dynamic world where relative timing between auditory and visual signals is highly variable and related signals may become temporally uncoupled due to a number of factors such as distance, luminance, attention and neural latencies (see e.g.^28^,^29^,^30^,^31^). Indeed, rapid recalibration to asynchronous audiovisual events would be extremely beneficial as the benefits of multisensory integration are greatest when the component signals are perceived simultaneously and decay with increasing asynchrony^9^,^10^,^11^,^32^. A relevant example would be the optimization of speech comprehension^8^, which is optimal when the audiovisual speech stimuli are perceived as simultaneous. By rapidly recalibrating to the first asynchronous audiovisual event in a speech stream (see^33^, for rapid recalibration with audiovisual speech stimuli), comprehension is likely to be optimized for the remainder of the stream. Moreover, rapid recalibration would reset instantly to a new speech stream received from a different distance and therefore with a different asynchrony.

As for the more prolonged manifestation of recalibration we observe, like the trial-by-trial effect this may serve to ‘delag’ prolonged and ongoing audio-visual delays that may arise from sustained exposure to audiovisual signals originating from a distant source. Whereas, this prolonged recalibration process may be beneficial in a context of sustained audio-visual lag, its lack of dynamic flexibility may in certain circumstances be problematic. For instance, a given asynchrony may be effectively realigned following sustained adaptation to that asynchrony, but for the duration of the decay period any temporal asynchronies in the opposite direction will be made even more asynchronous, with the maladaptive consequence that incoming signals may completely fail to activate multisensory mechanisms. Given these shortcomings it is worth considering an alternative possibility: that prolonged recalibration results from shifts in decisional criteria associated with judgments of simultaneity. Indeed, Yarrow and colleagues^34^ have recently argued that temporal recalibration may be entirely due to such criterion shifts, whereby subjects show an increased tendency to respond “synchronous” to trials with audiovisual lags of the same sign as the lag present during prolonged period of adaptation. In light of the findings described here and previously^23^, a tantalizing possibility may be therefore that the transient and sustained temporal recalibration observed here may in fact reflect different stages of the sensory-decisional process, with transient (trial-by-trial) recalibration mediated by shifts in temporal alignment of mechanisms associated with sensory timing mechanisms^35^ and prolonged exposure encouraging reweighting of sensory evidence at a higher-level decisional stage^34^.

To recap, we found evidence for inter-trial and prolonged temporal recalibration within a single experiment. Whereas prolonged recalibration causes a strong aftereffect that lasts approximately one minute, rapid recalibration varies from trial to trial, determined by the order of the audio-visual stimuli on the preceding trial. Moreover, we show that these recalibration effects are independent of each other and may therefore combine additively. Although the two effects are independent, it remains to be determined whether prolonged and inter-trial recalibration combine within a single mechanism or result from two distinct mechanisms. More research is required to clarify the underlying mechanism(s).

## Additional Information

**How to cite this article**: Van der Burg, E. *et al.* Audiovisual temporal recalibration occurs independently at two different time scales. *Sci. Rep.* **5**, 14526; doi: 10.1038/srep14526 (2015).
